# An Initiative to Reduce Preterm Infants Pre-discharge Growth Failure Through Time-specific Feeding Volume Increase

**DOI:** 10.1097/pq9.0000000000000366

**Published:** 2020-12-28

**Authors:** Sherman S. Chu, Heather O. White, Shannon L. Rindone, Susan A. Tripp, Lawrence M. Rhein

**Affiliations:** From the Department of Pediatrics, University of Massachusetts Memorial Medical Center, Worcester, Mass.

## Abstract

Very low birth weight infants often demonstrate poor postnatal longitudinal growth, which negatively impacts survival rates and long-term health outcomes. Improving extrauterine growth restriction (EUGR) among extremely premature infants has become a significant focus of quality improvement initiatives. Prior efforts in the University of Massachusetts Memorial Medical Center neonatal intensive care unit were unsuccessful in improving the EUGR rate at discharge.

**Methods:**

The primary aim of this initiative was to improve EUGR at discharge [defined as weight less than 10th percentile for postmenstrual age (PMA)] for infants born ≤32 0/7 weeks from a baseline of 25% to 20% by June 2019. We excluded all small for gestational age infants due to the limitation in the EUGR definition. A multidisciplinary team implemented evidence-based nutritional guideline changes using the Institute of Healthcare Improvement methods. The most notable change was the time-specific feeding volume advancement that increased the goal feeding volume between 31 0/7 and 34 0/7 weeks PMA from 150–160 to 170–180 milliliters per kilogram per day. The team monitored nutritional intake, weight, necrotizing enterocolitis (NEC), bronchopulmonary dysplasia (BPD), and length of stay (LOS).

**Results:**

The EUGR rate improved from 25% to 12% after initiation of increased time-specific, enteral feeding guidelines at 31–34 weeks PMA. NEC rate, BPD rate, and LOS remained unchanged throughout the initiative.

**Conclusions:**

By implementing a time-specific volume increase guideline from 31 0/7 to 34 0/7 weeks PMA, the EUGR rate improved from baseline of 25% to 12% without increasing NEC rate, BPD rate, and LOS.

## INTRODUCTION

Very low birth weight (VLBW) infants, defined as infants with a birth weight of <1,500 g, are at risk for multiple morbidities that can be significantly improved by adequate nutrition and growth.^1–4^ Unfortunately, VLBW infants typically demonstrate poor longitudinal growth patterns during their neonatal intensive care unit (NICU) hospitalization, leading to growth failure that persists after discharge.^3,5^ Weight less than the 10th percentile on the Fenton growth chart commonly defines growth failure in premature infants.^6,7^ Annually, the NICU discharges approximately 24% of premature infants born appropriate for gestational age (AGA) and 84% of premature infants born small for gestational age (SGA) with growth failure.^8^

Ensuring adequate postnatal growth is essential to improving long-term health outcomes in VLBW infants.^1–4^ Therefore, reducing extrauterine growth restriction (EUGR) has become a significant focus of quality improvement (QI) initiatives. Current strategies to enhance nutrition and minimize EUGR have included optimization of parental nutrition, varying schedules of feeding advances,^9^ and caloric supplementation.^10,11^ However, optimal timing and types of interventions to decrease EUGR rates remain incompletely understood.

Due to numerous studies that supported the benefit of early enteral feeding and advancement^12,13^ without an increased rate of necrotizing enterocolitis (NEC),^10–16^ the University of Massachusetts Memorial Medical Center (UMMMC) NICU implemented previous QI initiatives in 2016–2017 to reduce EUGR by introducing early enteral feeding and more rapid caloric fortification and volume advancement.^17^ However, despite achieving shorter time to full enteral feeds and decreased dependence on parenteral nutrition (PN) and central line utilization, the nutrition committee did not significantly improve the EUGR rate at discharge.

## AIMS

For this initiative, our team set the primary specific aim of reducing EUGR rate, defined as weight less than 10th percentile for postmenstrual age (PMA) at discharge, from baseline of 25% to 20% among AGA or large for gestational age (LGA) infants born less than or equal to 32 0/7 weeks by June 2019 (27 months). We defined AGA and LGA as 10th to 90th percentile in weight for gestational age (GA) and greater than 90th percentile in weight for GA on the Fenton revised growth chart,^7^ respectively. We targeted 90% compliance to the new data-driven enteral feeding guideline for all infants born <32 0/7 weeks GA by October 2018 (1 year from proposed guideline change). The team also monitored balancing measures of NEC, bronchopulmonary dysplasia (BPD), and length of stay (LOS).

## METHOD

### Context

The UMMMC is a 49-bed Level 3 NICU in Central Massachusetts. There are approximately 4,300 deliveries and 650 NICU admissions each year. On average, 100 of these admissions are VLBW infants. The UMMMC NICU team consists of multidisciplinary members from a rotating pool of 7 neonatologists, 11 neonatal nurse practitioners, 1 physician assistant, 3 neonatal fellows, resident physicians from multiple programs, 1 designated full-time nutritionist, and 150 staff nurses that participate in outlining and carrying out nutritional plans for NICU infants. To change practice and sustain change over time, therefore, would need to educate and obtain buy-in from 170 staff members.

### Nutritional Practice

**Fig. 1.**
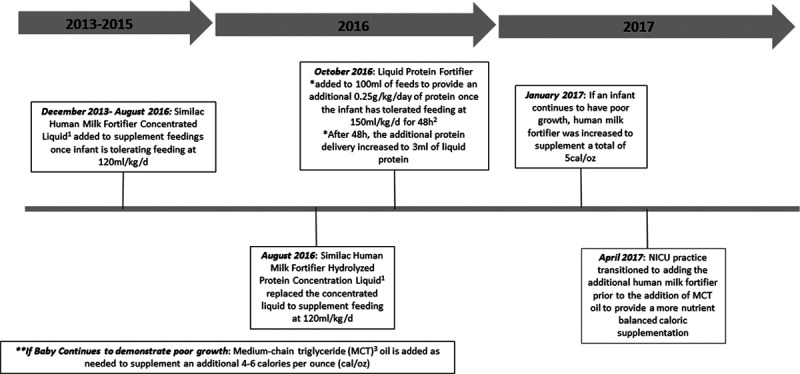
Nutrition guideline history: how to increase the caloric content of breast milk using human milk fortifier and liquid protein. ^1^Similac, Abbott Laboratories, Columbus, OH; ^2^Abbott Nutrition, Abbott Laboratories, Columbus, OH; ^3^Nestle Health Science, Nestle HealthCare Nutrition, Inc, Bridgewater, NJ.

The nutrition committee, consisting of a nutritionist, neonatologists, fellows, nurses, and a nurse educator, met monthly to review nutritional outcomes, guidelines, and practice barriers. The committee had been active in amending NICU guidelines based on NICU-specific growth outcomes and current literature. Guidelines recommended placing umbilical venous lines in infants born VLBW to provide PN while increasing enteral feeding volume as tolerated. We considered a total volume of 120 milliliters per kilogram per day (ml/kg/d) of enteral feeds as “full” enteral feeds, at which time we would remove the central line. Until October 2016, the nutrition enteral feeding advancement guidelines included adding human milk fortifier (Similac, Abbott Laboratories, Columbus, OH) to increase calories to 22 calories per ounce (cal/oz) after enteral volume had advanced to 100 ml/kg/d. Once enteral feeding reached the total volume, the guidelines recommended additional supplementation to 24 cal/oz. The prior QI initiative in 2016–2017 (decreasing time to full feeds) resulted in guideline changes including enteral feeding initiation at 24 hours of life, the faster daily advance of 20–30 ml/kg/d, and earlier caloric supplementation to 24 cal/oz once infants achieved an enteral volume of 80 ml/kg/d.^17^ The committee instituted guidelines to increase the caloric content of breast milk using a combination of human milk fortifier, liquid protein (Abbott Nutrition, Abbott Laboratories, Columbus, OH), and medium-chain triglyceride (MCT) oil (Nestlé Health Science, Nestlé Healthcare Nutrition, Inc., Bridgewater, NJ). The committee modified these supplements over time with changes in available products (Fig. 1).

In March of 2017, the nutrition committee implemented 2 changes in premixed starter PN concentration and administration practice that resulted in earlier higher nutrient delivery and faster advancement to target energy and protein. These modifications increased baseline protein provision from 1–2 to 3.2 gram per kilogram per day (g/kg/d) depending on total volume upon initiation. They decreased the time to goal lipid dose from 96–120 hours to 24–48 hours with routine triglyceride level monitoring. Since we encountered no significant issues with the practice change, we finalized and communicated the changes in April 2017 by introducing PN clinical practice guidelines as a reference tool, which detailed timing of initiation and advancement, and goal for PN components (macronutrients, electrolytes, and micronutrients).

Before 2016, the total number of infants born ≤32 0/7 weeks GA exposed to any human milk during their NICU stay was 53%. As breast milk feeding reduces the risk of NEC,^12,18,19^ in 2016, the nutrition committee reinforced the guideline of initiating donor breast milk (with parental consent) for all infants with birth weight <1,500 g when their own mother’s milk is unavailable. This practice change increased the human milk exposure rate to 94% with 57% human milk at discharge to home.

### Inclusions and Exclusions

We included all AGA and LGA infants admitted to the UMMMC NICU born ≤32 0/7 weeks GA, excluding those with congenital anomalies of the gastrointestinal tract. We included infants born SGA in the new guidelines but excluded them from outcome analyses consistent with the operational definition of EUGR with weight less than 10th percentile for PMA among infants who were previously AGA or LGA. We also excluded infants who died before discharge due to a lack of available discharge weight.

### Intervention

**Fig. 2.**
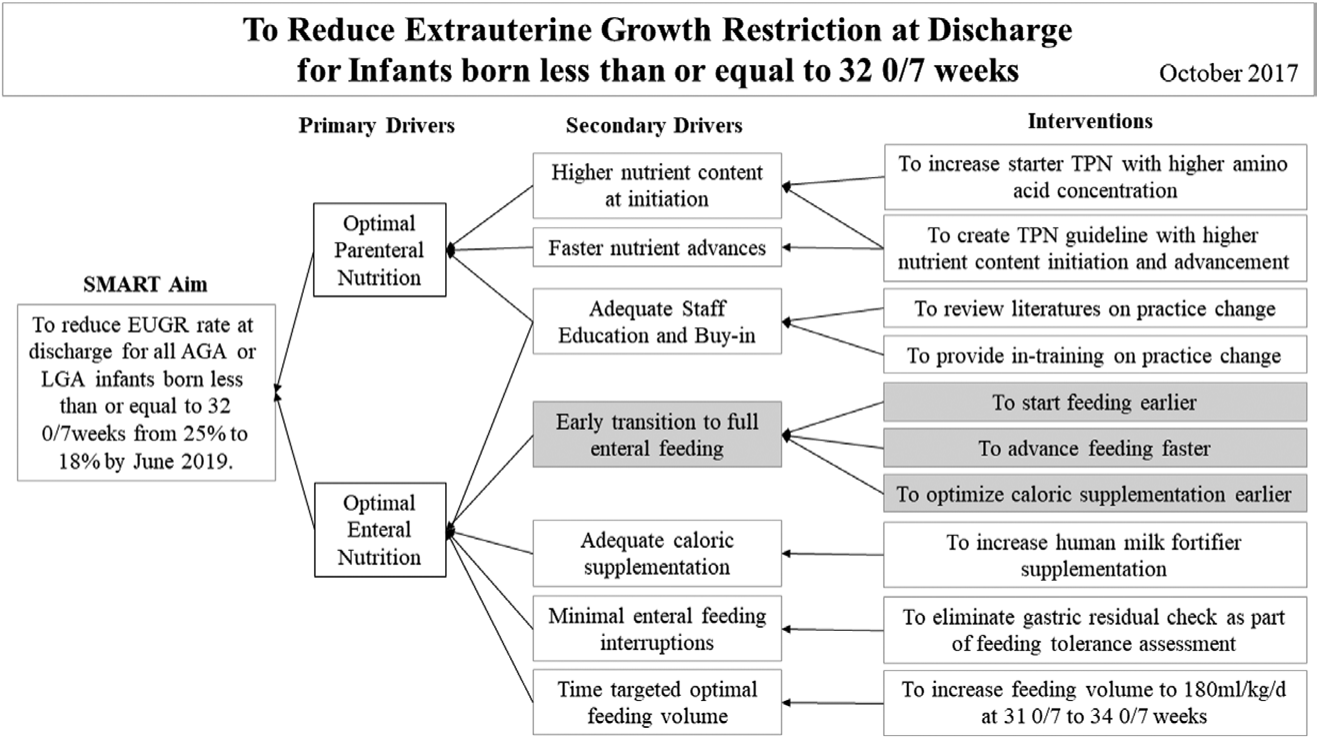
Key driver diagram to reduce EUGR at discharge for infants born less than or equal to 32 0/7 weeks. Gray indicates prior initiatives. TPN, total parenteral nutrition.

After the initiation of the enteral feeding guidelines, the nutrition committee evaluated the discharge EUGR rate by reviewing hospital growth data. We also evaluated the current literature to further optimize both parenteral and enteral nutritional practices throughout the NICU stay. We drafted a new specific aim and key driver diagrams to keep the initiative focused (Fig. 2). Although optimizing PN delivery was part of the improvement effort, the initiative focused on enteral feeding optimization since PN makes up a small proportion of nutritional support provided to an infant throughout the NICU stay.

In April 2017, planning the next set of Plan-Do-Study-Act (PDSA) cycles focused on identifying areas of enteral feeding guideline optimizations. In May 2017, we created and revised data collection tools to analyze the timing of growth failure for all infants born ≤32 0/7 weeks to determine potential factors associated with EUGR. We collected weight by PMA, feeding type and source, to determine if there was an association between breast milk type (maternal versus donor) or duration of donor breast milk use and the PMA at the time of growth failure. We tracked extrauterine growth patterns weekly to determine the timing that infants’ weight for age dropped below the 10th percentile. This analysis found no association between breast milk type and growth failure but instead found that most infants experienced weight decline below the 10th percentile between 32 0/7 and 35 0/7 weeks PMA. Based on these findings, the team set the goal to optimize enteral nutrition practice near the identified growth decline to address the potential higher nutritional needs at this time in their NICU course.

Once an infant has reached full enteral feeding, the methods to support growth include caloric fortification and total volume augmentation. The NICU had been routinely adding human milk fortifiers to enhance caloric and nutritional supplementation. Since we detected no complications from utilizing additional human milk fortifier amongst infants with poor growth, the nutrition committee changed the caloric supplement practice in April 2017. We recommended adding a fifth human milk fortifier per 100 ml to support growth and provide a more balanced caloric supplementation before supplementing with MCT oil. We then performed additional PDSA cycles to minimize feeding interruption due to gastric residual concerns, which led to the implementation of a feeding tolerance algorithm in August 2017.

**Fig. 3.**
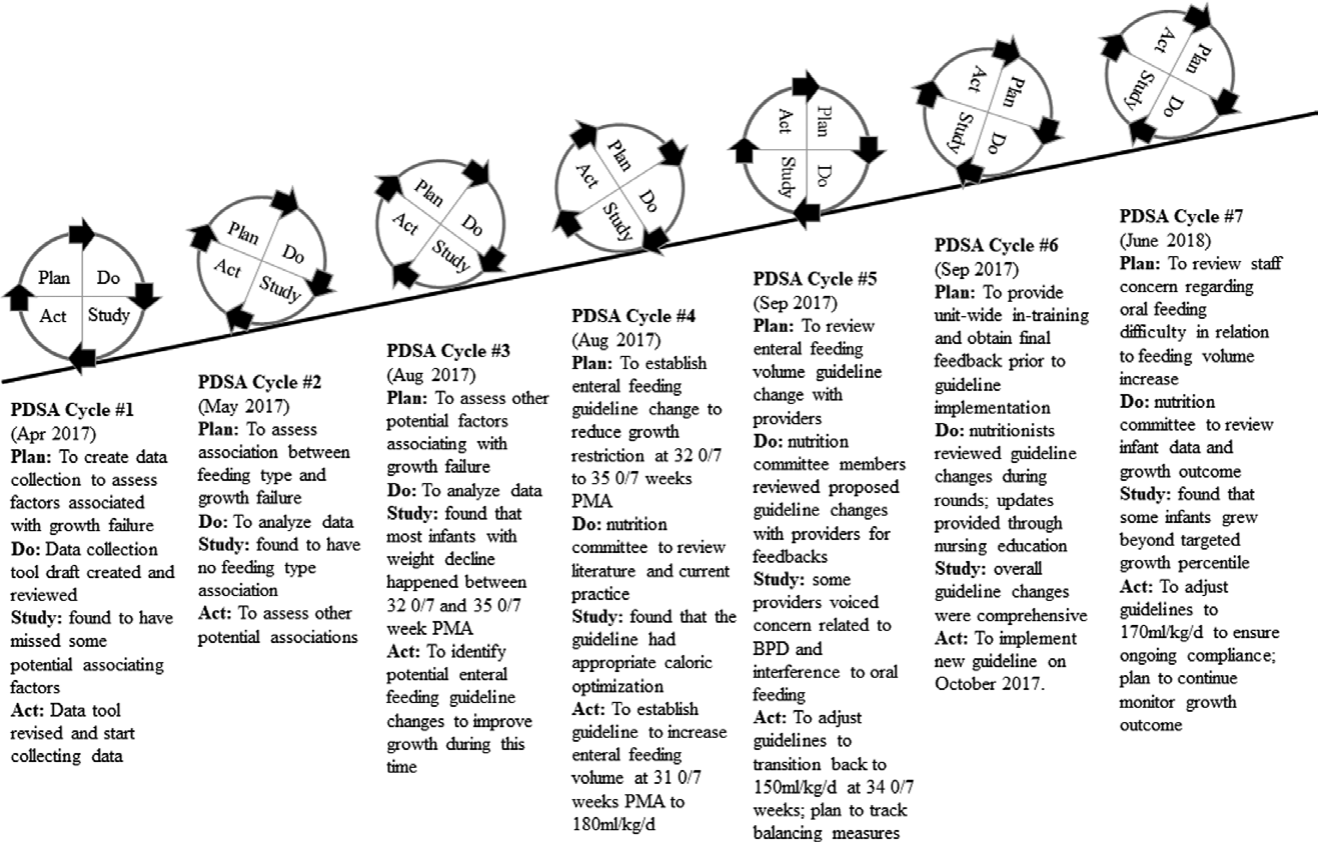
PDSA ramp cycles to establish enteral feeding guideline changes.

As possible links between higher osmolarity of feeds and increased intolerance and gastroesophageal reflux (GER) had been reported,^20,21^ the current initiative then focused on increasing total enteral volume without significantly altering enteral feed composition. We developed guidelines to increase total feeding volume for all infants born ≤32 0/7 weeks GA from 150–160 ml/kg/d to 170–180 ml/kg/d starting at 31 0/7 weeks PMA. Even though the period of growth decline occurred until 35 0/7 weeks PMA, the guideline ended volume increase at 34 0/7 weeks PMA to then resume the routine volume target of approximately 150 ml/kg/d. The guideline reduced feeding volume at 34 0/7 weeks PMA back to baseline to minimize the potential effect on feeding development associating with hunger cues. Subsequent PDSA cycles focused on guideline revision and staff education and feedback, resulting in guideline implementation in October 2017 (Fig. 3).

Within several months of the increased enteral feeding volume guideline change, staff informally raised concern to committee members that the new guidelines might be associated with feeding intolerance and increased LOS. To ensure staff satisfaction and ongoing guideline compliance, the team reevaluated the enteral feeding guidelines and considered potential guideline modifications. Since the review of preliminary growth outcomes demonstrated that some infants grew beyond the targeted growth percentile, the team decided to decrease the volume goal between 31 0/7 and 34 0/7 weeks PMA from 180 to 170 ml/kg/d. The nutrition committee submitted a revised guideline for review and implemented the changes on October 1, 2018, after proper staff education.

### Measures

We performed a prospective review of hospital records as part of standard quality of care monitoring with weekly updated measures. We gathered prior QI initiatives^17^ and prior Vermont Oxford Network (VON) hospital reports for baseline data. Thus, we defined NEC and BPD based on the VON operational definitions.^22^ We categorized the measures into outcome, process, and balancing measures.

Rate of EUGR was the global aim outcome measure, defined as the number of infants previously born AGA or LGA discharged with weight less than 10th percentile for PMA per total number. Compliance with the volume increase guidelines was the process measure. It was defined as the number of occurrences of enteral feeding volume increase starting at 31 0/7 weeks and weight adjusting weekly to maintain the same volume goal per weight until 34 0/7 weeks PMA. The volume increase target was 180 ml/kg/d from October 2017 to October 2018, and to 170 ml/kg/d starting October 2018. To ensure no unwanted complication from the change in guidelines, the nutrition committee closely monitored NEC rate, BPD rate, and LOS as balancing measures. We identified NEC as at least 1 clinical sign (bilious gastric aspirate or emesis, abdominal distension, or occult or gross blood in stool without obvious anal fissure) and 1 radiographic finding (pneumatosis intestinalis, hepatobiliary gas, or pneumoperitoneum).^22^ We classified BPD as a supplemental oxygen requirement at 36 0/7 weeks PMA or home discharge.^22^ LOS was defined as the duration of hospital stay from birth to home discharge.

### Data Analysis

We utilized process control charts to track changes related to the interventions during the project period. We used QIMacros (KnowWare International, Inc, Denver, CO) add-on in Microsoft Excel (Microsoft Corp., Redmond, WA) to prepare the charts. We plotted the centerline with baseline values. We then shifted the line once we identified special cause variations (8 points above or below the centerline).

### Ethical Considerations

The implementation of evidence-based interventions to standardize the nutritional management of infants born ≤32 0/7 weeks GA and admitted to the UMMMC NICU was the basis of this QI work. QI team members assessed infant medical records within the scope of normal practice and responsibilities to improve unit practice without accessing personal health information outside UMMMC. The UMass Institutional Review Board determined that this initiative was not human subject research and did not require IRB approval.

### Process and Outcome Reporting

The team used the checklist provided in the SQUIRE publication guidelines^23^ as a framework to report the process and outcomes of this QI effort. After thorough consideration, we omitted the components considered not applicable to the initiative.

## RESULTS

**Fig. 4.**
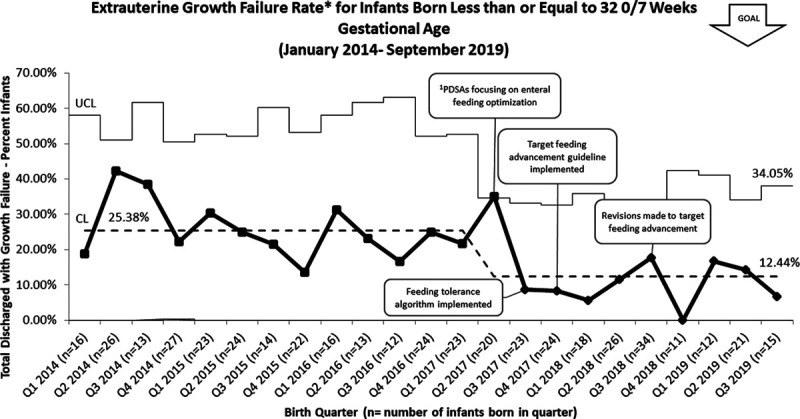
P chart of extrauterine growth failure rate at discharge at UMMMC for infants born less than or equal to 32 0/7 weeks GA. ^1^PDSA cycle timeline, please refer to Figure 2 for full list of PDSA cycles included and their initiation dates.

**Fig. 5.**
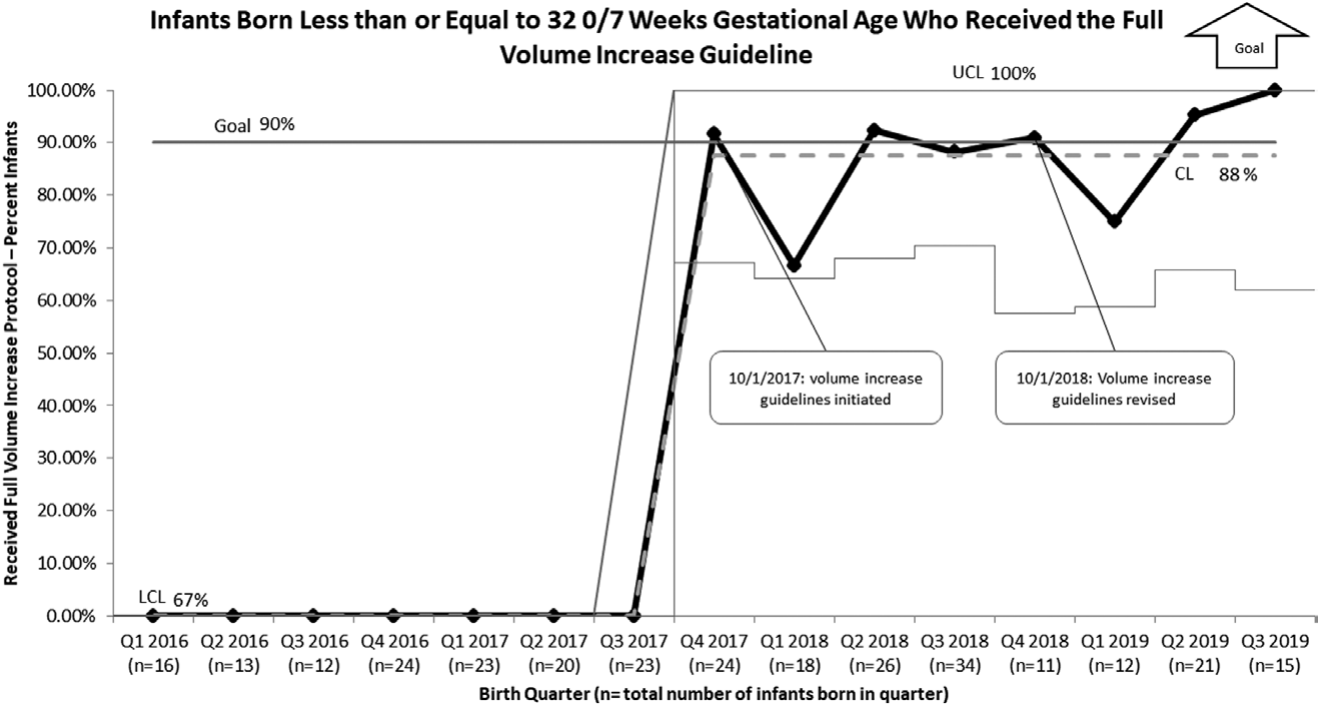
P chart of volume increase guideline compliance for all infants born less than or equal to 32 0/7 weeks GA.

The UMMMC NICU EUGR rate at discharge notably decreased after initiation of the time-specific increased volume guidelines. The baseline EUGR rate at UMMMC was 25% before the start of this initiative. After successful implementation, the EUGR rate decreased to 12%, surpassing the global aim of EUGR rate reduction to 20% (Fig. 4). After the most recent change of decreasing the volume goal to 170 ml/kg/d, we maintained the improvement in the EUGR rate for 3 subsequent quarters. Overall compliance with the volume increase guidelines was 87% for all eligible infants (Fig. 5). In addition to the compliance rate, post hoc we randomly selected 10% of infants from the pre-implementation (January 2016 to September 2017) and post-implementation (September 2017 to September 2019) cohorts and found an increase in median total feeding volumes(147 and 163 ml/kg/d, respectively).

**Fig. 6.**
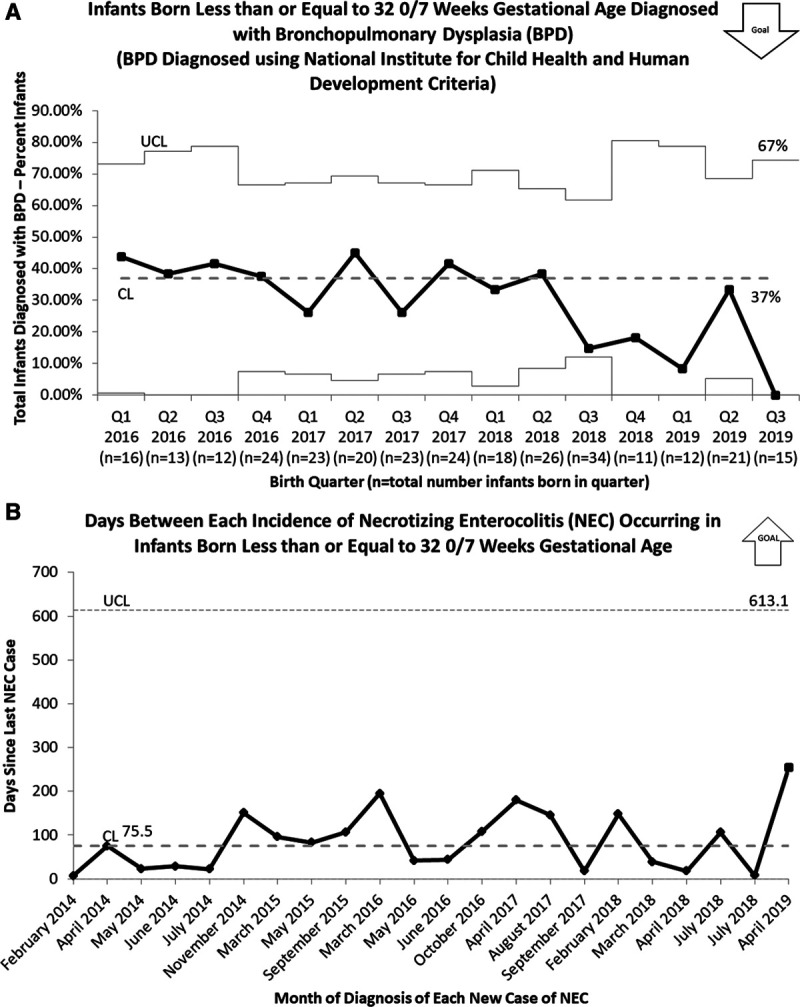
Balancing measures for feeding volume increase. A, P chart of infants born less than or equal to 32 0/7 weeks GA diagnosed with BPD at UMMMC. B, T chart displaying duration (days) between each incidence of NEC occurring in infants born less than or equal to 32 0/7 weeks.

There was no increase found amongst balancing measures. BPD rates for eligible infants did not increase but showed a trend toward improvement after the initiative (Fig. 6). Per UMMMC data collection for the VON collaborative, NEC rates remained constant at 4%, with an average of 89 days between individual NEC cases (Fig. 6). We omitted the actual dates of NEC occurrence on the graph to deidentify the data further. Despite the perception that there was an increase in the LOS during the change in guidelines to feeding volume of 180 ml/kg/d, there was no significant change noted with LOS maintained at 60 days.

## DISCUSSION

The time-specific increase-volume-feeding guideline initiative appreciably decreased the EUGR rate in infants born ≤32 0/7 weeks GA. We were successful in reaching beyond the identified global aim of a 5% reduction of discharge EUGR rates, from 25% to 12% and is notably lower than previously reported rates of VLBW infants born AGA.^8^ The proposed volume increase guideline was well received by providers, especially when it was decreased to 170 ml/kg/d. We achieved 87% compliance with the volume increase guideline. Our team determined that the 13% noncompliance rate was likely related to clinical concerns of feeding tolerance issues common to premature infants, and was appropriate for this initiative.

Prior recommendations have described growth failure occurring early in the NICU course, leading to guidelines focusing on the transition from parental to enteral nutrition, rather than later course interventions.^9–11^ In contrast, no previous literature described the time-specific drop in weight percentile found among the AGA infants between 32 0/7 and 35 0/7 weeks PMA in this initiative. This time-specific growth restriction and its link to developmental milestones such as temperature regulation with incubator weaning and transition to oral feeding from gavage feeding are potential areas for future research.

The high rate of guideline compliance supported the association between the time-specific increased feeding volume practice and the decrease EUGR rate. The weight gain beyond the expected growth curve found in a subset of infants ceased the need for supplementing caloric density with liquid protein/MCT oil, which appeared to have improved their feeding tolerance. The improvement in linear growth would have been a significant finding, as poor linear growth is associated with poor neurodevelopmental outcomes.^24^ However, the lack of length and body mass index monitoring due to the non-standardized length measurement practice limited the growth outcome interpretation. Also, the EUGR definition of our current initiative limited assessment of the growth of our SGA infants. The analysis and monitoring of z-score changes and improved length measurement will be the focus of future nutrition committee meetings.

The possible relationship between NEC and variation in feeding practice continues to be an area of concern. Some evidence suggests an association between hyperosmolality of feeds and NEC.^25^ As optimization of caloric supplementation had been the standard nutritional practice, providing additional nutritional support through increased feeding volume was a logical next step. The baseline NEC rate did not change after the initiation of the guidelines with no prior studies demonstrating any link between increased enteral volumes and NEC. However, it is generally difficult to establish any apparent association with NEC due to its overall low incidence.

We identified BPD as a balancing measure due to previously described concern regarding GER and feeding volumes, and their potential relationship with oral aversions, apnea episodes, and BPD in premature infants.^26,27^ Multiple previous studies failed to demonstrate a relationship between GER and apnea episodes^28–30^ or BPD.^31,32^ Although previous literature suggested concerns of the potential exacerbating effects of increased volume on patent ductus arteriosis^33^ and its association with BPD,^34,35^ the BPD rate decreased throughout the initiative. LOS has remained unchanged along with the lack of an increase in comorbidities like NEC and BPD.

Issues common to other QI initiatives, such as the inability to compare groups in the same timeframe and concurrent QI initiatives that might have affected the outcomes (ie, BPD reduction initiatives), limited the interpretation of this QI initiatives outcomes. Other concurrent nutritional changes made along with the time-specific volume increase may have contributed to the EUGR reduction at discharge. However, the 87% compliance with time-specific nutrition guidelines was likely the major contributor to success due to its problem-focused approach.

## CONCLUSIONS

A time-specific volume increase guideline at 31 0/7–34 0/7 weeks PMA significantly contributed to the reduction of EUGR at discharge in VLBW infants. The increased volumes during this time did not result in increased rates of NEC, BPD, or LOS. This simple guideline is applicable and easily generalizable to other NICUs.

## DISCLOSURE

The authors have no financial interest to declare in relation to the content of this article.
